# RhoA suppresses pseudorabies virus replication in vitro

**DOI:** 10.1186/s12985-023-02229-2

**Published:** 2023-11-15

**Authors:** Xin-Man Li, Shi-Ping Wang, Jin-Yuan Wang, Ting Tang, Bo Wan, Lei Zeng, Jiang Wang, Bei-Bei Chu, Guo-Yu Yang, Jia-Jia Pan

**Affiliations:** 1 College of Veterinary Medicine, Henan Agricultural University, Zhengzhou, 450046 China; 2 Key Laboratory of Animal Biochemistry and Nutrition, Ministry of Agriculture and Rural Affairs of the People’s Republic of China, Zhengzhou, 450046 China; 3 Key Laboratory of Animal Growth and Development of Henan Province, Zhengzhou, 450046 China; 4 Ministry of Education Key Laboratory for Animal Pathogens and Biosafety, Henan Agricultural University, Zhengzhou, 450046 China; 5 College of Animal Science and Technology, Henan University of Animal Husbandry and Economy, Zhengzhou, 450047 China

**Keywords:** RhoA, Actin cytoskeleton, Viral replication, Pseudorabies virus

## Abstract

The porcine pseudorabies virus (PRV) is one of the most devastating pathogens and brings great economic losses to the swine industry worldwide. Viruses are intracellular parasites that have evolved numerous strategies to subvert and utilize different host processes for their life cycle. Among the different systems of the host cell, the cytoskeleton is one of the most important which not only facilitate viral invasion and spread into neighboring cells, but also help viruses to evade the host immune system. RhoA is a key regulator of cytoskeleton system that may participate in virus infection. In this study, we characterized the function of RhoA in the PRV replication by chemical drugs treatment, gene knockdown and gene over-expression strategy. Inhibition of RhoA by specific inhibitor and gene knockdown promoted PRV proliferation. On the contrary, overexpression of RhoA or activation of RhoA by chemical drug inhibited PRV infection. Besides, our data demonstrated that PRV infection induced the disruption of actin stress fiber, which was consistent with previous report. In turn, the actin specific inhibitor cytochalasin D markedly disrupted the normal fibrous structure of intracellular actin cytoskeleton and decreased the PRV replication, suggesting that actin cytoskeleton polymerization contributed to PRV replication in vitro. In summary, our data displayed that RhoA was a host restriction factor that inhibited PRV replication, which may deepen our understanding the pathogenesis of PRV and provide further insight into the prevention of PRV infection and the development of anti-viral drugs.

**Supplementary Information:**

The online version contains supplementary material available at 10.1186/s12985-023-02229-2.

## Introduction

The Pseudorabies virus (PRV), also called suid herpesvirus 1, belongs to the *Herpesviridae* family, *α-herpesvirinae* subfamily and *varicellovirus* genus [1]. PRV is an enveloped virus that harbors a linear double-stranded DNA genome of approximate 150 kb in length and encodes at least 70 gene products [1]. PRV infection causes severe reproductive, respiratory and neurological diseases in pigs that leads to huge economic losses to the global swine industry [1–3]. Besides, PRV has a wide infection of animals, such as mouse, sheep, mink, cattle and dog, resulting in severe clinical symptoms and death [4–6]. Although widespread use of the Bartha-K61 vaccine in controlling PRV, it remains a destructive infectious disease in many countries. More importantly, several recent reports implicate that PRV variant strain can be detected in humans and lead to endophthalmitis and encephalitis [7–9]. These reports suggest that PRV infection remains difficult to eradicate and has become a potential public health threat. Therefore, it is necessary to develop potent vaccine and drugs to prevent and control the transmission of PRV.

Viruses depend on host cell organelles and molecular components for cell entry and propagation. The host cytoskeleton is one of the first cellular systems hijacked by viruses in order to invade host cells and produce progeny virions [10–12]. Small GTPases are a class of proteins widely involved in intracellular signal pathways governing different processes, from cytoskeletal and organelle organization to gene transcription and intracellular trafficking [13, 14]. Small GTPases are also known to be involved in infection by several intracellular pathogens, including viruses, bacteria and protozoan parasites. Rho GTPases participate in distinct viral life cycle by regulating actin cytoskeleton dynamics [15–17]. In this study, we recapitulate the function and mechanisms by which PRV manipulate the host cell during infection, focusing on the role of RhoA and actin cytoskeleton.

As one member of small Rho-GTPase family, RhoA is a key regulator of cytoskeleton system. The extracellular signal such as virus infection can activate the Rho-GTPase and its downstream effectors to form complicated signal network that leads to the dynamics changes of the cytoskeleton, which in turn regulates the viral life cycle [15]. It has been reported that RhoA and its downstream effectors were activated during certain virus infection [18–21]. For example, human parainfluenza virus V protein promoted virus growth by inducing RhoA activation and RhoA-induced F-actin formation [20]. Classical swine fever virus activated RhoA/ROCK and Rac1/Cdc42/PAK pathways in the early stage of infection, which helped virus entry into host cells by regulating the dynamic changes of the F-actin [21]. However, the vaccinia virus F11 protein promoted viral release and spread by inhibiting RhoA-mDia signaling and by modulating the actin cytoskeleton dynamics [22, 23]. It has been previously shown that RhoA is key modulator that facilitates filopodia formation during Kaposi’s sarcoma-associated herpesvirus entry [24, 25]. Human cytomegalovirus and herpes simplex virus type-1 have been shown to exploit RhoA and RhoB isoforms or other Rho family members, facilitating crucial steps of viral infection [26–30]. Besides, the US3 protein of PRV triggered RhoA phosphorylation to reorganize the actin cytoskeleton [31]. These reports implicated that RhoA pathway may be involved in the PRV infection.

In this study, we examined the roles of RhoA and actin cytoskeleton during PRV infection. By using specific inhibitor and genetic knockdown, we revealed that inhibition of RhoA promoted PRV proliferation. Conversely, activation of RhoA by chemical drug or genetic overexpression inhibited PRV infection. Altogether, our findings suggested that RhoA was a host restriction factor that inhibited PRV infection, whose anti-viral activity might be related with the regulation of actin cytoskeleton.

## Materials and methods

### Materials

The Cell Counting Kit-8 (CCK-8) was ordered from Yeasen BioTechnologies co, Ltd. (Shanghai, China). Anti-GAPDH was purchased from Proteintech Group, Inc. (Chicago, USA). Anti RhoA was purchased from Novus Biologicals (Colorado, USA). Antiserum against PRV glycoprotein gB was generated by immunization of mice with purified recombinant gB. Goat anti-Mouse IgG, goat anti-Rabbit IgG, Alexa-Fluor-488-conjugated goat anti-mouse were purchased from Thermo Fisher Scientific (Shanghai, China).

### Cells and viruses

Porcine kidney epithelial PK-15 (CCL-33, ATCC) were cultured in monolayers at 37℃ under 5% CO_2_ in DMEM medium (10566-016, GIBCO) supplemented with 10% FBS (10099141C, GIBCO), 100 U/mL penicillin and 100 µg/mL streptomycin sulfate (15070063, GIBCO). The virulent PRV isolate QXX (PRV-QXX) and the recombinant PRV strain of PRV-GFP, derived from PRV Hubei strain with TK gene replaced by GFP expression cassette from the pEGFP-N1 plasmid, was kindly donated by Professor Bei-bei Chu from the College of Veterinary Medicine, Henan Agricultural University.

### Cell viability assay

PK-15 cells were seeded into 96-well plates with 1 × 10^4^ cells/well. On the next day, the medium was changed to DMEM/10% FBS supplemented with various concentrations of certain chemical drugs for the indicated time. Cell viability was determined with CCK-8 according to the manufacturer's instructions. The absorbance at 450 nm was detected with a microplate reader (Awareness Technology Inc, FL, USA).

### Western blotting

Whole-cell lysates were extracted with lysis buffer (50 mM Tris–HCl, pH 8.0, 150 mM NaCl, 1% Triton X-100, 1% sodium deoxycholate, 0.1% SDS, 2 mM MgCl_2_) supplemented with protease and phosphatase inhibitors (Roche, Mannheim, Germany). The protein concentrations in the lysates were quantified with a BCA Protein Assay Kit (DingGuo, Beijing, China), detected with a microplate reader (Awareness Technology Inc, FL, USA). Protein samples were separated by SDS-PAGE, transferred to nitrocellulose membranes (Millipore, Billerica, MA, USA), and incubated in 5% non-fat milk (Sangon, Shanghai, China) for 1 h at room temperature. The membranes were incubated with primary antibody overnight at 4℃and then incubated with horseradish-peroxidase- conjugated secondary antibody (Thermo Fisher Scientific, Shanghai, China) for 1 h at room temperature. Immunoblotting results were visualized using Luminata Crescendo Western HRP Substrate (Millipore) on GE AI600 imaging system (Boston, MA, USA).

### Quantitative real-time PCR (RT-qPCR)

Total RNA was isolated by using Trizol Reagent (Takara, Shiga, Japan) and subjected to cDNA synthesis with the PrimeScript™ RT Reagent Kit (Takara). RT-qPCR was performed in triplicate by using SYBR Premix Ex Taq (Takara, Shiga, Japan), and data were normalized by the level of *GAPDH* expression in each individual sample. Melting curve analysis indicated formation of a single product in all cases. The 2^−ΔΔCt^ method was used to calculate relative expression changes. For quantification of PRV genome copy number, PCR product of 187 bp from the gene of PRV glycoprotein H (gH) was cloned into pGEM-T vector. Serial tenfold dilutions of this plasmid were used to construct a standard curve. The total number of PRV genomic equivalents was determined by comparison with the standard curve. Primers used for RT-qPCR are presented in Table 1.

**Table 1:** Primers used for gene cloning and RT-qPCR analysis

Name	Sequence (5′-3′)
Sus-GAPDH-Fw	GAAGGTCGGAGTGAACGGAT
Sus-GAPDH-Rv	CATGGGTAGAATCATACTGAACA
Sus-RhoA-Fw	GATGAGCACACAAGGCGTGA
Sus-RhoA-Rv	TGCTGAACACTCCATGTACC
Sus-PRV-gB-Fw	GGCATCGCCAACTTCTTCC
Sus-PRV-gB-Rv	CCTCGTCCACGTCGTCCTC
Sus-PRV-TK-Fw	GGCGTACTGGCGCACTCTG
Sus-PRV-TK-Rv	ATGTCCCCGACGATGAAGC
Sus-PRV-gH-Fw	CTCGCCATCGTCAGCAA
Sus-PRV-gH-Rv	GCTGCTCCTCCATGTCCTT

### RNA interference (RNAi)

Negative control and RhoA-specific siRNAs were designed with BLOCK-iT™ RNAi Designer (Life Technologies, Carlsbad, CA) and commercially synthesized (Genepharma, Shanghai) (Table 2). 20 pmol/well siRNAs were transfected into PK-15 cells using Lipofectamine RNAiMAX Transfection Reagent (Invitrogen, Grand Island, NY), according to the manufacturer’s instructions. For a transfection in six-well plate, PK-15 cells were grown to 70–80% confluence before transfection. siRNAs and Lipofectamine RNAiMAX were diluted with DMEM (Gibco, Grand Island, NY) and incubated at room temperature for 5 min. Lipid-siRNA complexes were mixed and incubated for an additional 20 min and added drop-wise to cells. The knockdown efficiency of RhoA was determined by RT-qPCR and Western blot at 48 h post-transfection. Each assay was performed in triplicate.

**Table 2:** Sequence of siRNA used for gene knockdown

Name	Sequence (5′-3′)
Negative control-S	UUCUCCGAACGUGUCACGUTT
Negative control-AS	ACGUGACACGUUCGGAGAATT
RhoA-Sus-428-S (siRhoA-1)	GUCUUUAGCAAGGACCAAUTT
RhoA-Sus-428-AS (siRhoA-1)	AUUGGUCCUUGCUAAAGACTT
RhoA-Sus-526-S (siRhoA-2)	CAGGUAGAGUUGGCUUUGUTT
RhoA-Sus-526-AS (siRhoA-2)	ACAAAGCCAACUCUACCUGTT

### Plasmid and transfection

The coding sequence of porcine RhoA gene was amplified from the cDNA of PK-15 cells with the specific primers. The sequences of the primers were: FLAG-Fw, 5′-CAAGCTTGCGGCCGCGAATTCATGGCTGCCATCAGGAAGAA-3′ and FLAG-Rv, 5′-CCTCTAGAGTCGACTGGTACCTCACAAGACAAGGCACCCAGA-3′. The PCR product was cloned into p3 × FLAG-CMV-10 (Sigma-Aldrich) to generate FLAG-RhoA. Transfection of plasmid was performed with Lipofectamine®3000 Transfection Reagent (Invitrogen, Grand Island, NY), according to the manufacturer’s instructions. Each assay was performed in triplicate.

### Flow cytometry

For viral proliferation assays, cells were infected with recombinant virus expression the GFP reporter gene for 24–48 h and digested with trypsin–EDTA (25200072, GIBCO). Then, cells were collected by centrifugation at 1000 g for 5 min and suspended in phosphate-buffered saline (PBS). The percentage of GFP positive cells was measured by flow cytometry on CytoFLEX (Beckman, Atlanta, GA, USA). All data were analyzed by CytExpert software.

### Immunofluorescence assay (IFA)

Cells grown on glass coverslips (Thermo Fisher Scientific) were fixed with 4% paraformaldehyde for 10 min. After washing with PBS, the cells were permeabilized with PBS containing 0.1% Triton X-100 for 10 min and then incubated in PBS/10% FBS for 60 min. And then incubated with PBS containing 10% FBS with the primary antibody for 1 h at room temperature. After washing with PBS, the cells were further incubated with fluorescent secondary antibodies (1:500, Invitrogen) in PBS/10% FBS for 1 h. After the cells were washed three times with PBS and then were labeled with phalloidin (1:200, Invitrogen) in PBS/1% BSA for 20 min. The cells were finally washed in PBS and mounted in ProLong Diamond with DAPI (#P36971, Invitrogen). Images were captured on a Zeiss LSM 800 microscope.

### Plaque forming unit assay (PFU)

The virus suspension was serially diluted by tenfold serial dilutions for 4 times, and then inoculated on the monolayer PK-15 cells in 24-well plates (3 × 10^5^ cells per well). The viruses were adsorbed at 37 ℃ for 1 h, and changed with 1% FBS DMEM for 4–7 days. The supernatant was discarded, and 4% PFA was added to fix the cells at room temperature for 30 min. Then, the cells were stained with 1% crystal violet for 30 min, and the plaques and non-shedding cells were observed after 30 min of water immersion. After drying, the number of plaques under the microscope was counted.

### Statistical analysis

All data were obtained from at least three independent experiments for quantitative analyses and are expressed as means ± standard errors of the means. Western blot signal intensity was analyzed using Image J software. All data were analyzed using the Prism 8.0.2 software (GraphPad, CA, USA). All statistical analyses were performed with one-way analysis of variance (ANOVA). Significant differences relative to the corresponding controls were accepted at *P < 0.05, **P < 0.01 and ***P < 0.001.

## Results

### Inhibition of RhoA promotes PRV infection

**Fig. 1:**
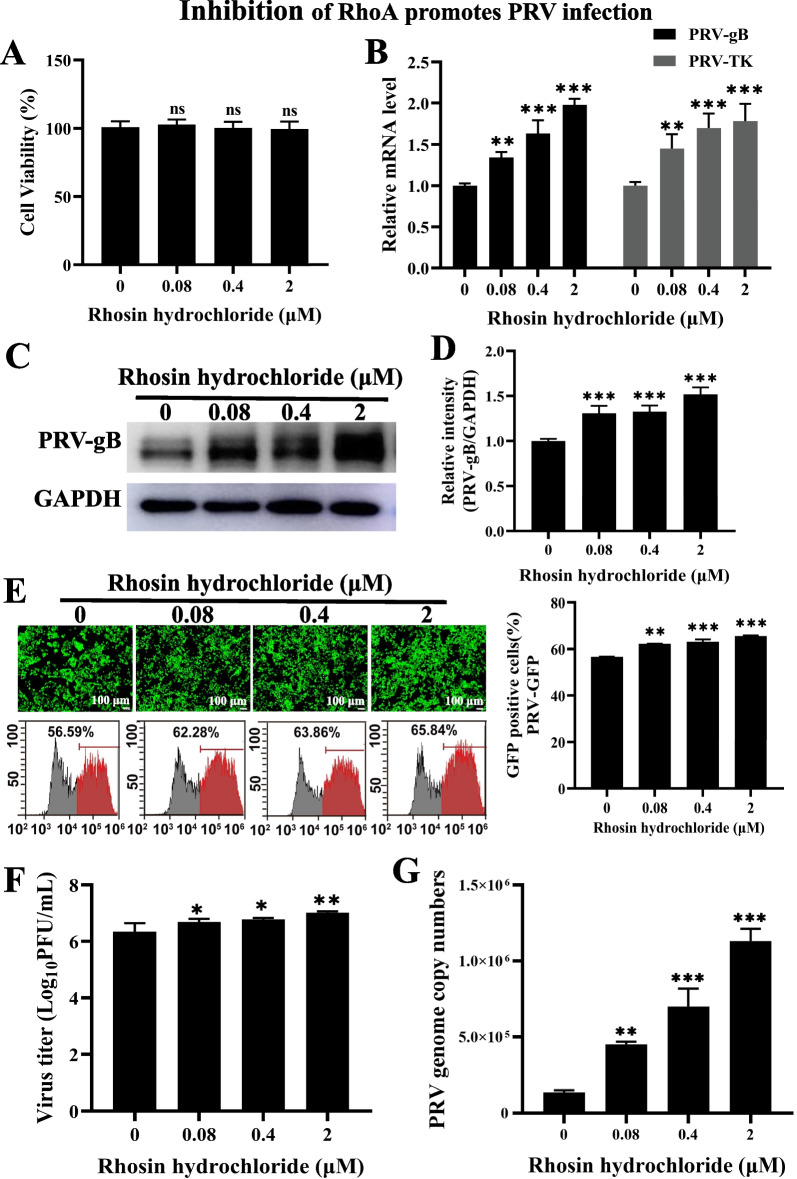
Inhibition of RhoA promotes PRV infection. **A** PK-15 cells were treated with Rhosin (0–2 µmol/L) for 24 h. CCK-8 assays were then used to determine the cell viability (%). **B** PK-15 cells were pretreated with various concentrations of Rhosin for 4 h and infected with PRV-QXX (MOI = 0.1) for 24 h, expression analysis of PRV *gB* and *TK* mRNA levels using RT-qPCR. *GAPDH* served as a loading control. **C** Immunoblotting analysis of PK-15 cells treated as in **B** and infected with PRV-QXX (MOI = 1) with the indicated antibodies. **D** Gray value analysis of C using Image J software. **E** PK-15 cells treated as in **B** and infected with PRV-GFP (MOI = 0.01) for 24 h, then cells were observed under fluorescence microscope and the GFP-positive cells were measured by flow cytometry. Scale bar, 100 μm. **F** PK-15 cells treated as in **B** and infected with PRV-QXX (MOI = 0.1), viruses were harvested with three freeze–thaw cycles, and the viral titer was determined by PFU assay. **G** PK-15 cells treated as in **B** and infected with PRV-QXX (MOI = 0.1), determination of the PRV genome copy number based on PRV-gH. All the data are shown as mean ± SD based on three independent experiments

We aimed to determine whether RhoA was involved in PRV infection. Rhosin hydrochloride (Rhosin) specifically inhibited RhoA activity and RhoA-mediated cellular function without affecting Cdc42 or Rac1 signaling activities [32]. Firstly, for the normal development of follow-up experiments, we performed cell viability assay to examine the cytoxicity of Rhosin by CCK-8. As shown in Fig. 1A, 0.08–2 µmol/L of Rhosin concentration was chosen for the optimal inhibition of RhoA activity with nontoxic on PK-15 cells. The mRNA levels of PRV *gB* and *TK* (thymidine kinase) in Rhosin-treated cells were significantly higher than those in control cells (Fig. 1B). This indicated that the transcription of PRV genes was promoted by Rhosin. PRV-gB protein expression was enhanced in a Rhosin dose-dependent manner (Fig. 1C, D), which is consistent with PRV *gB* mRNA level. Rhosin treatment resulted in an increase of PRV-GFP proliferation, as indicated by fluorescent microscope and flow cytometry analysis of GFP-positive cells (Fig. 1E). We next detected the multiplication of PRV progeny virus in response to Rhosin using a viral titer assay. As shown in Fig. 1F, Rhosin significantly promoted the production of PRV progeny virus. Besides, PRV genome copy numbers were also increased in Rhosin treated cells (Fig. 1G). These data demonstrated that inhibition of RhoA benefited PRV infection.

### RhoA knockdown enhances PRV infection

**Fig. 2:**
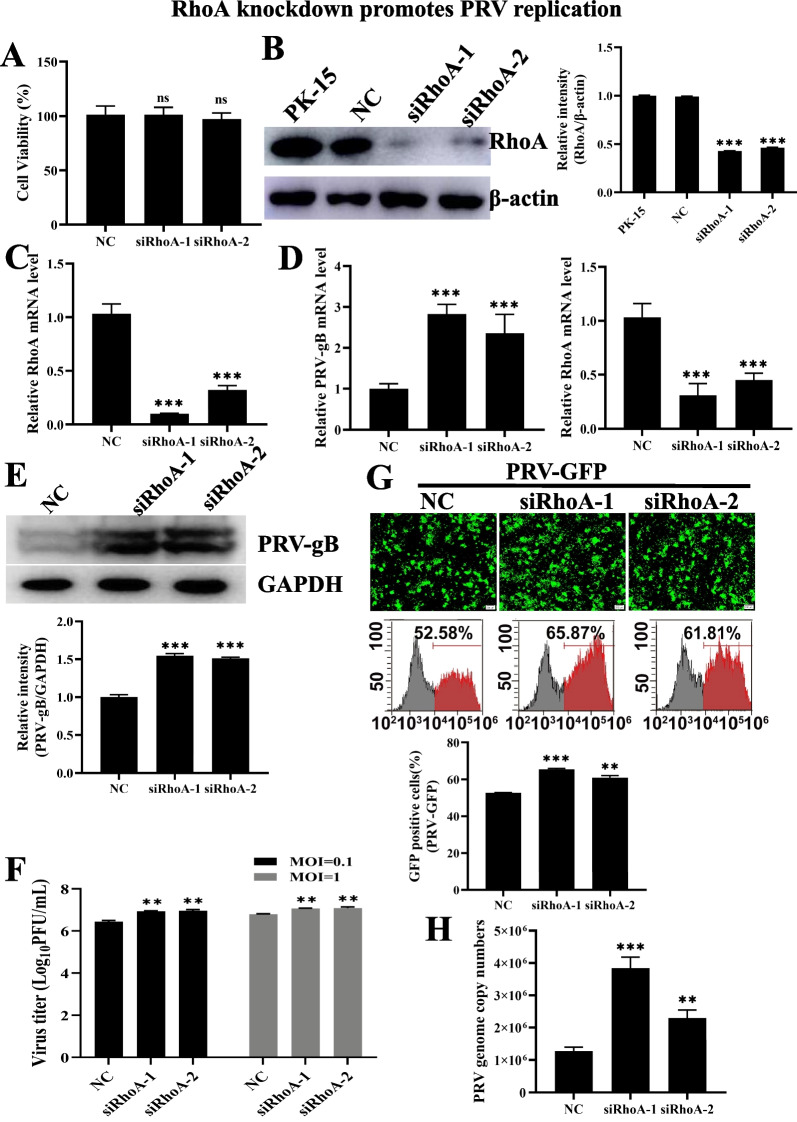
RhoA knockdown enhances PRV infection. **A** Cell proliferation assay of PK-15 transfected with RhoA siRNA and control siRNA for 24 h. **B** Immunoblotting analysis of whole cell extracts from PK-15, NC, siRhoA-1 and siRhoA-2 PK-15 cells with antibodies against RhoA. β-actin served as a loading control. Gray value analysis of PRV-gB intensity by Image J software. **C** RT-qPCR analysis of *RhoA* mRNA in NC, siRhoA-1 and siRhoA-2 PK-15 cells. *GAPDH* served as a loading control. **D** RT-qPCR analysis of PRV *gB* and *RhoA* mRNA in NC, siRhoA-1 and siRhoA-2 PK-15 cells infected with PRV-QXX (MOI = 0.1) for 24 h. *GAPDH* served as a loading control. **E** Immunoblotting analysis of whole cell extracts from NC, siRhoA-1 and siRhoA-2 PK-15 cells infected with PRV-QXX (MOI = 1) for 24 h with antibody against PRV gB. GAPDH served as a loading control. Gray value analysis of PRV-gB intensity by Image J software. **F** PK-15 cells treated as in **B** and infected with PRV-QXX (MOI = 0.1 and 1), viruses were harvested with three freeze–thaw cycles, and the viral titer was determined by PFU assay. **G** Fluorescent microscopy and flow cytometry analysis of PRV-GFP (MOI = 0.01) proliferation in NC, siRhoA-1and siRhoA-2 PK-15 cells for 24 h. Scale bar, 200 μm. **H** NC, siRhoA-1 and siRhoA-2 PK-15 cells infected with PRV-QXX (MOI = 1) for 24 h, determination of PRV genome copy number based on PRV-gH. Data are shown as mean ± SD based on three independent experiments

To further validate the role of RhoA in PRV *infection, we investigated PRV infection by genetic knockdown of RhoA* with siRNA. We performed a CCK-8 assay to identify whether RhoA knockdown influenced cell viability. The cell viability of two independent siRNAs specifically targeting RhoA (siRhoA-1 and siRhoA-2) were almost the same as control cells (Fig. 2A). The efficiency of RhoA knockdown was analyzed by western blot (Fig. 2B). siRhoA-1 and siRhoA-2 showed significant knockdown efficiency, proven by RT-qPCR with housekeeping gene GAPDH (Fig. 2C). RT-qPCR and western blot analysis showed that PRV gB gene transcription and protein expression were both increased by RhoA knockdown (Fig. 2D, E). In addition, the viral titer was then quantified by plaque assays. As shown in Fig. 2F, knockdown of RhoA increased viral progeny production (MOI = 0.1 and 1). Next, we infected siRNA-RhoA and mocked PK-15 cells with PRV-GFP and performed fluorescent microscopy and flow cytometry analysis, which showed that knockdown of RhoA increased the PRV-GFP positive cells (Fig. 2G). Besides, we infected cells with PRV-QXX and performed a viral genome quantitation assay (Fig. 2H). These data suggested that knockdown of RhoA promoted PRV replication in PK-15 cells, which was identical with the results of RhoA inhibitor treatment. The above results indicated that RhoA might be a host restriction factor for PRV infection.

### Activation of RhoA by Narciclasine inhibits PRV infection

**Fig. 3:**
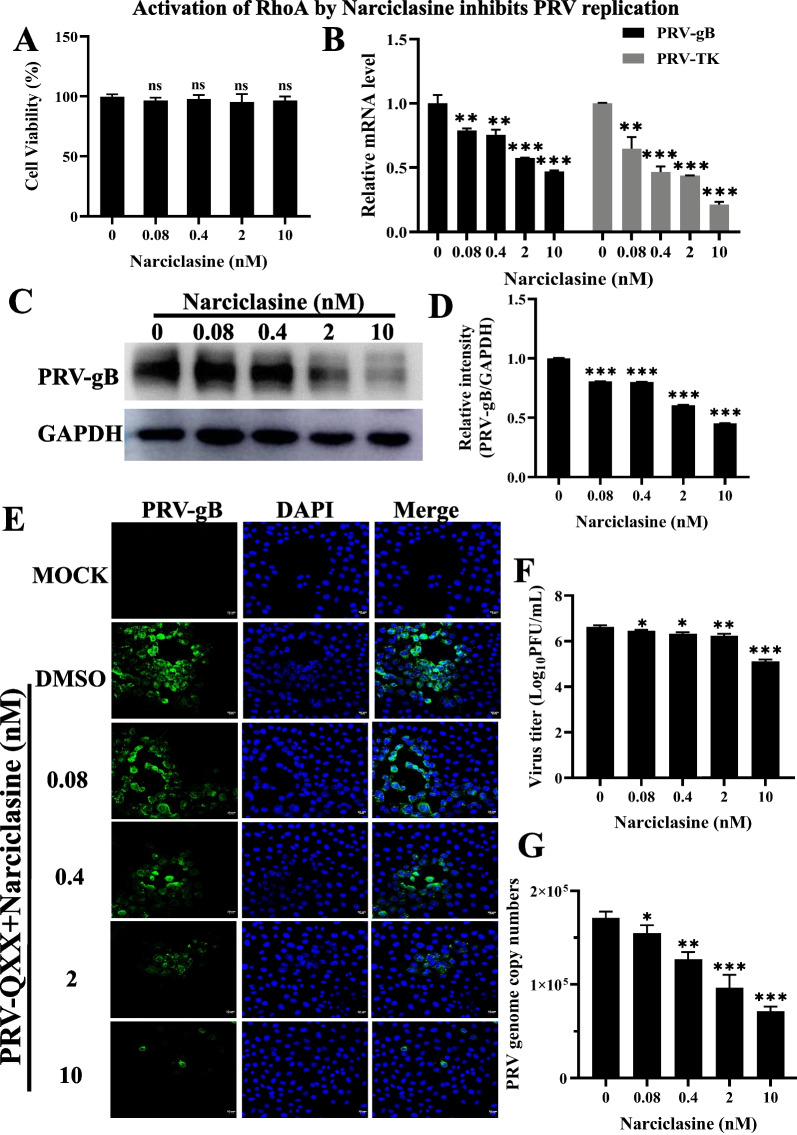
RhoA agonist inhibits PRV replication. **A** PK-15 cells were treated with Narciclasine (0–10 nmol/L) for 24 h. CCK-8 assays were then performed to determine the cell viability (%). **B** PK-15 cells were pretreated with Narciclasine at indicated concentrations for 4 h and infected with PRV-QXX (MOI = 0.1) for 24 h, expression analysis of PRV gB and TK mRNA levels using RT-qPCR. GAPDH served as a loading control. **C** Immunoblotting analysis of PK-15 cells treated as in **B** and infected with PRV-QXX (MOI = 1) for 24 h with the anti-PRV-gB antibody. GAPDH served as a loading control. **D** Gray value analysis of PRV gB using Image J software. **E** After treatment as described in B, the cells were fixed and stained with antibody of PRV gB (in green) and the nuclei were stained with DAPI. The fluorescence was measured by Zeiss LSM 800 microscope. Scale bar, 10 μm. **F** PK-15 cells treated as in **B**, viruses were harvested with three freeze–thaw cycles, and the viral titer was determined with PFU assays. **G** PK-15 cells treated as in **B** and infected with PRV-QXX (MOI = 0.1) for 24 h, determination of PRV genome copy number based on PRV-gH. All the data are shown as mean ± SD based on three independent experiments

To confirm the negative role of RhoA in PRV replication, we utilized an agonist of RhoA, Narciclasine, which was isolated from Hippeastrum puniceum. As a plant growth modulator, Narciclasine modulates the Rho/Rho kinase/LIM kinase/Cofilin signal pathway, greatly increasing RhoA-GTPase activity as well as inducing actin stress fiber formation in a RhoA-dependent manner [33]. Besides, it reported that Narciclasine showed antiviral activity against dengue virus and Zika virus [34]. Firstly, cell viability assay was performed to measure the cytoxicity of Narciclasine in PK-15 cells. As shown in Fig. 3A, the concentration of Narciclasine used in this study is nontoxic. Next, we examined the effect of Narciclasine in PRV-QXX replication by immunoblotting analysis of PRV glycoprotein gB, and RT-qPCR analysis the transcription of PRV *gB* and *TK* genes in PK-15 cells. The mRNA levels of PRV *gB* and *TK* in Narciclasine-treated cells were significantly lower than those in control cells, and showed a dose-dependent manner (Fig. 3B). This indicated that Narciclasine restricted the transcription of PRV genes. PRV gB protein level was lower in Narciclasine-treated cells, which also showed a dose-dependent manner (Fig. 3C, D). Meanwhile, similar dose-dependent inhibition of PRV infection was observed by the IFA assay (Fig. 3E). We then verified the inhibitory effect of RhoA agonist on PRV infection by a viral titer assay. PK-15 cells were treated with Narciclasine (0.08–10 nmol/L) and infected with PRV-QXX (MOI = 0.1) for 24 h. Multiplication of the PRV progeny virus decreased with an increased concentration of Narciclasine (Fig. 3F). Besides, the genomic copy number of PRV-QXX was also decreased in Narciclasine-treated cells (Fig. 3G). These data demonstrated that RhoA played a negative role in PRV infection.

### Overexpression of RhoA inhibits PRV infection

**Fig. 4:**
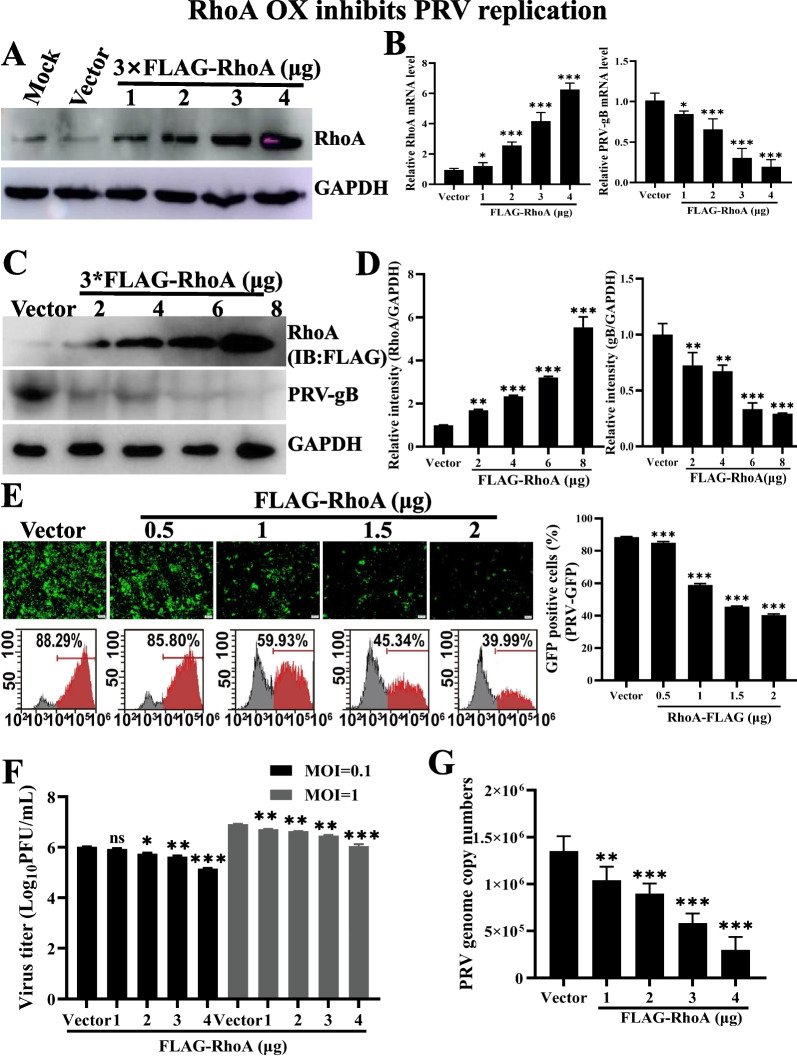
Overexpression of RhoA inhibits PRV replication. **A** Immunoblotting analysis of whole cell extracts from PK-15 cells expressing vector or FLAG-RhoA with antibody against RhoA. GAPDH served as a loading control. **B** RT-qPCR analysis of PRV *gB* and *RhoA* mRNA level in PK-15 cells expressing vector or FLAG-RhoA infected with PRV-QXX (MOI = 0.1) for 24 h. *GAPDH* served as a loading control. **C** Immunoblotting analysis of whole cell extracts from PK-15 cells expressing vector or FLAG-RhoA infected with PRV-QXX (MOI = 1) for 24 h with antibodies against PRV gB and FLAG. GAPDH served as a loading control. **D** Gray value analysis of **C** using Image J software. **E** Fluorescent microscopy and flow cytometry analysis of PRV-GFP (MOI = 0.01) proliferation in PK15 cells expressing vector or FLAG-RhoA for 36 h. Scale bar, 200 μm. **F** PFU assay in vector or FLAG-RhoA PK-15 cells infected with PRV-QXX (MOI = 0.1 or 1) for 24 h. **G** PK-15 cells treated as in **B** and infected with PRV-QXX (MOI = 1) for 24 h, determination of PRV genome copy number based on PRV-gH. Data are shown as mean ± SD based on three independent experiments

To further confirm the role of RhoA in PRV infection, we overexpressed the RhoA gene in PK-15 cells by FLAG-RhoA. The efficiency of RhoA overexpression was measured via Western blotting and RT-qPCR, respectively. Results showed that RhoA was successfully expressed compared with the control group (Fig. 4A, B). These results revealed that PK-15 cells expressing porcine RhoA were successfully established. Next, we explored the effect of RhoA overexpression on PRV infection in PK-15 cells. PK-15 cells expressing FLAG-RhoA generated less PRV gB mRNA than control cells did (Fig. 4B). Meanwhile, we examined the effect of RhoA overexpression on PRV gB expression by immunoblotting analysis. As shown in Fig. 4C, D, PRV gB protein expression was decreased due to the expression FLAG-RhoA. In addition, we infected control (vector) and RhoA-overexpressing cells with PRV-GFP and fluorescent microscopy and flow cytometry assay showed that GFP positive cells were fewer in RhoA-overexpressing cells than in control cells, suggesting that RhoA overexpression inhibited PRV-GFP infection (Fig. 4E). The viral titer and viral genomic copy number were then quantified by plaque assays and RT-qPCR, respectively. As shown in Fig. 4F, overexpression of RhoA reduced viral progeny production (MOI = 0.1 and 1). Besides, the genomic copy number of PRV-QXX was also decreased in FLAG-RhoA cells (Fig. 4G). Taken together, these results implicated that RhoA was a host restrictive factor that inhibited PRV infection.

### Actin cytoskeleton polymerization contributes to PRV replication in vitro

**Fig. 5:**
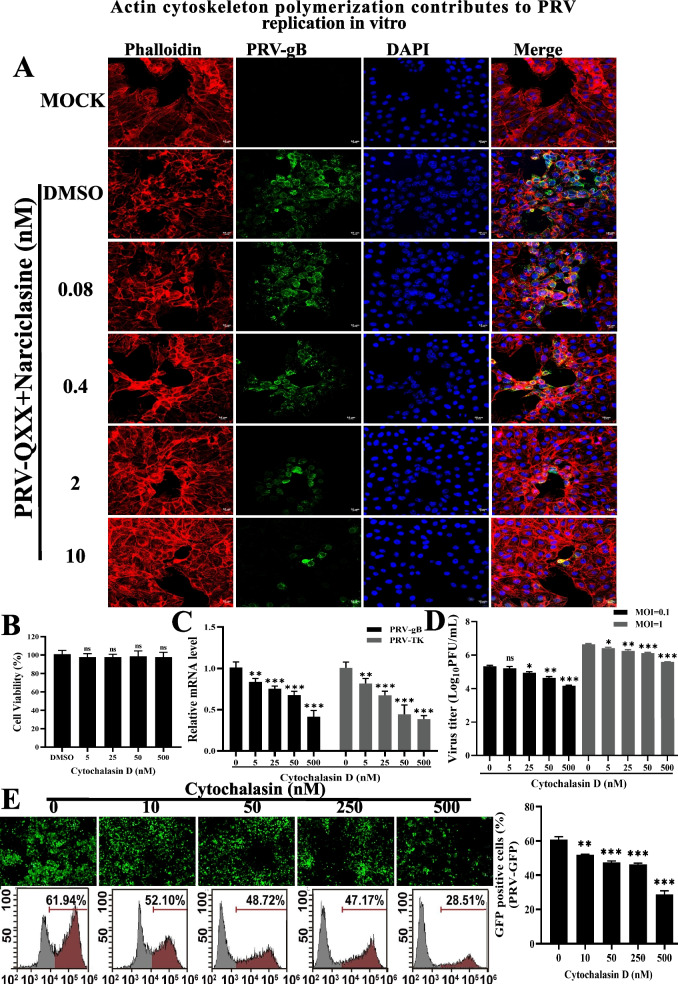
Actin cytoskeleton polymerization contributes to PRV replication in vitro. **A** PK-15 cells were treated with different concentrations of Narciclasine for 4 h, infected with PRV-QXX (MOI = 0.1) for 24 h with the above concentrations of compound. The cells were fixed with 4% PFA, stained with anti-gB antibody for viral glycoprotein gB, phalloidin for F-actin and DAPI for nucleus. Fluorescence was analyzed by confocal microscopy. Scale bar, 10 μm. **B** PK-15 cells treated with cytochalasin D (0–500 nmol/L) for 24 h, CCK-8 assays were then performed to determine the cell viability (%). **C** RT-qPCR analysis of PRV *gB* and *TK* mRNA expression in cytochalasin D (0–500 nmol/L) treated cells infected with PRV-QXX (MOI = 0.1) for 24 h. *GAPDH* served as a loading control. **D** PK-15 cells treated as in C and infected with PRV-QXX (MOI = 0.1 or 1) for 24 h, then viruses were harvested with three freeze–thaw cycles, and the viral titer was determined with PFU assays. **E** Fluorescence microscopy and flow cytometry analysis of PK-15 cells infected with PRV-GFP (MOI = 0.01) for 24 h after treatment with cytochalasin D (0–500 nmol/L). Scale bar, 100 μm. Data are shown as mean ± SD based on three independent experiments

IFA assay showed that PRV infection disrupted host cell actin stress fiber formation (Fig. 5A, second row), which is consistent with previous report [35]. However, the disruption of stress fiber formation by PRV infection was reorganized when RhoA was activated by Narciclasine, which in turn inhibition of PRV proliferation (Fig. 5A, row third to six). Therefore, we speculated that RhoA-induced actin structural reorganization may be involved in the negative regulation of PRV replication. Furthermore, in order to explore the effect of actin cytoskeleton on PRV replication, PK-15 cells were pretreated with different concentrations of cyto D for 4 h before infection with PRV-QXX (MOI = 0.1). RT-qPCR analysis showed that treatment with cyto D decreased the mRNA level of PRV *gB* and *TK* gene in a concentration-dependent manner (Fig. 5C) with no significant effect on cell viability (Fig. 5B), indicating that cyto D treatment inhibited PRV infection, which is consistent with previous report [36]. We next detected the multiplication of PRV progeny virus in response to cyto D using a viral titer assay. As shown in Fig. 5D, cyto D significantly inhibited the production of PRV progeny virus. Additionally, infection of PK-15 cells with PRV-GFP after treatment with serial concentration of cyto D, fluorescence microscope and flow cytometry assay showed that PRV-GFP positive cells were fewer in cytochalasin-treated cells than in control cells, suggesting that disruption of actin filament inhibited PRV-GFP infection (Fig. 5E). Take Figs. 3, 4 and 5 into account, overexpression or activation of RhoA induced the reorganization of actin cytoskeleton and prohibited PRV replication, whereas disruption of actin by cyto D treatment also hampered PRV infection. In all, these data indicated that the dynamics change of actin cytoskeleton is important for PRV infection, which might be regulated by RhoA.

## Discussion

As parasitic pathogens, many viruses depend on host cell organelles and molecular components for cell entry and proliferation. The host cytoskeleton is widely manipulated during all steps of virus life cycle, including viral attachment and entry, transportation to the replication site and release of progeny virions to the extracellular environment [37–39]. Actin is one of the most abundant cytoskeletons in eukaryotes. A variety of viruses have been found to utilize diverse approaches to regulate actin cytoskeletons to create a suitable microenvironment for effectively infection [40–42]. For example, dengue serotype 2 (DENV2) infection induced the reorganization of actin filaments and the small GTPase Rac1 was involved in replication cycle of DENV2 via regulation of the actin cytoskeleton [43]. ZIKV infection induced actin filaments rearrangement, which in turn benefited ZIKV infection [44]. Actin alterations have also been associated with changing of fusogenicity of cells during viral infection [45]. Therefore, a natural and intact cytoskeletal structure is required for efficient virus infection [40, 46]. However, PRV infection disturbed the natural actin cytoskeletal structure by disrupting the formation of actin filaments, which facilitated its replication. In this study, IFA assay showed that PRV infection disrupted host actin filament dynamics in PK-15 cells (Fig. 5A, second lane), which was consistent with previous report [35]. Moreover, the disruption of actin filaments with cyto D inhibited PRV proliferation, as demonstrated by a decreased of PRV *gB* and *TK* gene level and PRV-GFP positive cells (Fig. 5C, E). Besides, cyto D significantly inhibited the production of PRV progeny virus (Fig. 5D). These findings indicated that actin cytoskeleton was involved in the PRV infection. Cyto D is known to destabilize actin dynamics via inhibiting the polymerization of subunit by binding to the plus-ends of the actin filaments. Under our experimental condition, the disruption of actin filaments with cyto D inhibited PRV proliferation, indicating that the polymerization of actin cytoskeleton was important for virus infection.

Rho GTPases are nucleotide-dependent molecular switches that are involved in multiple cellular function, especially act as master regulators of actin cytoskeleton organization [47, 48]. RhoA, Cdc42 and Rac1 are the most widely researched and the best understood members of this family of proteins, which function as a bridge connecting the cell surface receptors to regulate the actin cytoskeleton and participate in the invasion of various viruses into host cells [21, 49, 50]. Rho GTPases can be activated by different virus infection and this alteration plays an essential role in the viral replication cycle [18, 22, 31, 49]. Despite the critical roles of Rho GTPases and their regulation in the rearrangement of cytoskeleton in the process of viral replication, many relevant Rho GTPase regulatory proteins remain uncharacterized for their effect in PRV infection. In this study, we explored the effects of RhoA and actin cytoskeleton during PRV infection. As shown in Figs. 1 and 2, inhibition of RhoA by small molecule chemical inhibitor and siRNA promoted PRV replication, as evident by increasing in the viral mRNA and protein synthesis, genome copy numbers and progeny virus yield. On the contrary, overexpression of RhoA or activation of RhoA by Narciclasine inhibited PRV proliferation (Figs. 3 and 4). These data suggested that RhoA was a host restriction factor that inhibited PRV infection. In addition, our results showed that disruption of actin filaments with cyto D elicited similar effects on PRV proliferation with RhoA (Fig. 5). Together, these results indicated that RhoA and the polymerization of actin cytoskeleton were important for PRV infection.

Rho GTPases are well established as mediators in the endocytosis of many viruses such as herpesvirus, paramyxovirus and Ebola virus [17, 51, 52]. It reported that Japanese encephalitis virus could regulate actin cytoskeleton by activating RhoA and Rac1, which in turn promoted virus entry into human neurons through caveolin-mediated endocytosis [53]. Besides, HIV-1 Env-guided entry was supported by a Filamin A-RhoA-ROCK axis and Arp2/3 complex, both of which were commonly involved in actin cytoskeletal reorganization [54]. During PRV infection, the expression of RhoA increased in the early phase (0–6 h.p.i) and decreased in late phase (12–24 h.p.i) (Additional file 1: Fig. S1). Considering that virus entry is one of the essential steps in the virus life cycle, it is necessary to further study the role of RhoA in PRV attachment and internalization.

Rho GTPases regulate cytoskeleton remodeling by regulating a wide variety of downstream substrates. ROCK and mDia are downstream effectors of RhoA GTPase. ROCK is a serine/threonine kinase and has been shown to phosphorylate the myosin-binding subunit of myosin phosphatase as well as myosin light chain [55]. The emerging evidences have established a link between virus infection and Rho/ROCK/Myosin and Rho/mDia signaling pathway, although the precise mechanism underlying regulation of virus replication by this signaling remains elusive. For example, porcine sapovirus infection induced early activation of the RhoA/ROCK/MLC pathway in polarized LLC-PK cells, which resulting in virus entry into cells [49]. However, Rock1 translocated to the nucleus and inhibited human cytomegalovirus propagation [56], and Rho-ROCK-MLC contractility signaling pathway resisted sendai virus fusion with host cell that may provide a physical barrier to host cells against viral fusion [57]. Despite the novel insights, the present study was unable to determine the downstream molecules and mechanisms underlying the PRV life cycle; thus, prospective studies will focus on these associations.

Viruses rapidly develop drug-resistant variants, so developing host-target antiviral therapeutics is a major challenge. Rho GTPases have been implicated in diverse cellular functions and are potential diagnostic biomarkers and/or therapeutic targets. RhoA-derived peptides have been shown to have antiviral activity against human respiratory syncytial virus, human immunodeficiency virus-1 and human parainfluenza virus-3 [58–60]. Besides, RhoA specific agonist Narciclasine had antiviral activity against dengue virus and Zika virus [34]. Herein, we showed that Narciclasine inhibited PRV infection. Taken together, the results of the present study suggest that RhoA may exert an antiviral effect against PRV, and these functions are at least partially mediated by the polymerization of actin cytoskeleton. These results may contribute to better understanding of the importance of the RhoA signal pathway in virus infection and encourage the investigation of future translational application of combining PRV strains with RhoA drug or other cytoskeleton modifying agents.

## Conclusions

In summary, we report here that the RhoA and actin cytoskeleton play important roles in PRV infection in porcine epithelial cell. Inhibition of RhoA promotes PRV proliferation, whereas activation of RhoA restricts PRV infection. Besides, our results also show that the polymerization of actin cytoskeleton is important for PRV infection. Overall, these results elucidate that RhoA and actin cytoskeleton play important roles in PRV infection. Insight into the virus and host interaction not only contributes to our understanding of viral pathogenesis, but also shed light on the development of novel anti-viral drugs.

### Supplementary Information

**Additional file 1: Supplementary figure 1**. Expression of RhoA in response to PRV infection.

## Data Availability

All data and materials generated for this study are included in the article.
