# Supplementary figures and images for: RhoA suppresses pseudorabies virus replication in vitro
Source: Virol J. 2023 Nov 15;20:264. doi: 10.1186/s12985-023-02229-2 (PMC10652432; doi:10.1186/s12985-023-02229-2)

# Supplementary figure 1. Expression of RhoA in response to PRV infection

## A

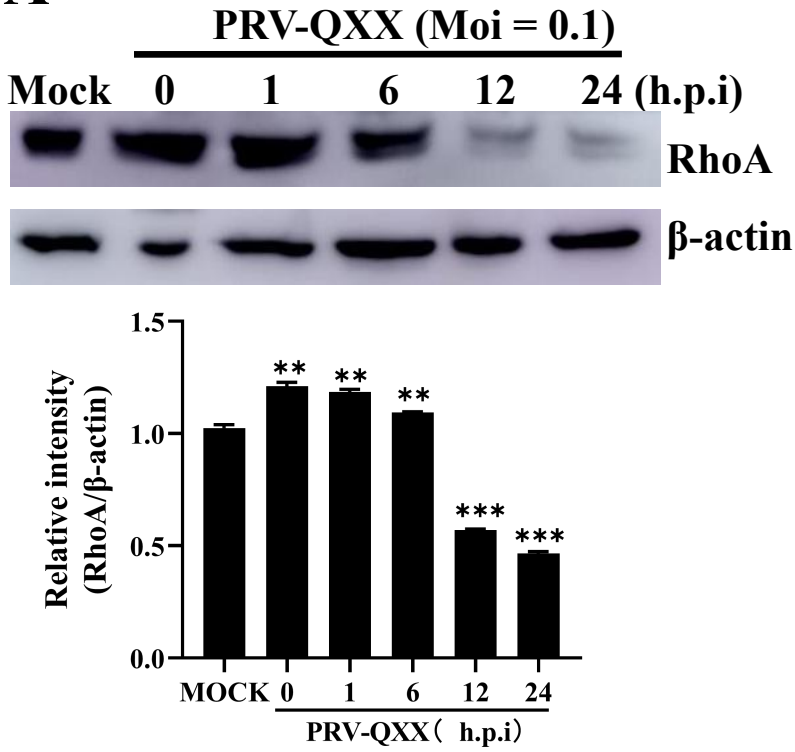

## B

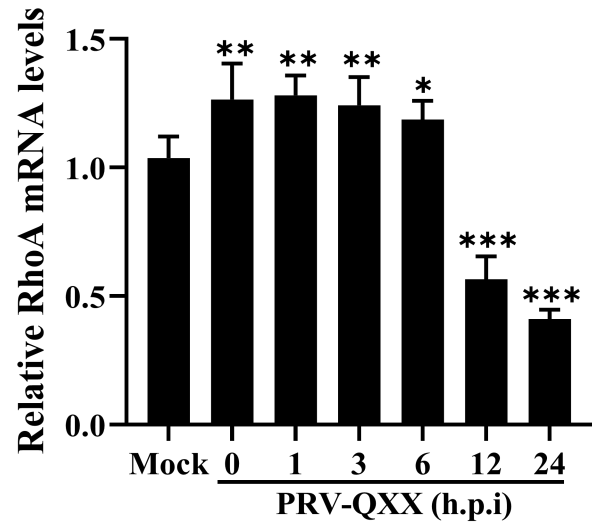

Supplement: Supplementary file 1 — Additional file 1: Supplementary figure 1. Expression of RhoA in response to PRV infection. [file 12985_2023_2229_MOESM1_ESM.pdf]
